# Bivalent IAP antagonists, but not monovalent IAP antagonists, inhibit TNF-mediated NF-*κ*B signaling by degrading TRAF2-associated cIAP1 in cancer cells

**DOI:** 10.1038/cddiscovery.2016.46

**Published:** 2017-01-16

**Authors:** Y Mitsuuchi, CA Benetatos, Y Deng, T Haimowitz, SC Beck, MR Arnone, GS Kapoor, ME Seipel, SK Chunduru, MA McKinlay, CG Begley, SM Condon

**Affiliations:** 1 TetraLogic Pharmaceuticals Corporation, 343 Phoenixville Pike, Malvern, PA 19355, USA

## Abstract

The inhibitor of apoptosis (IAP) proteins have pivotal roles in cell proliferation and differentiation, and antagonizing IAPs in certain cancer cell lines results in induction of cell death. A variety of IAP antagonist compounds targeting the baculovirus IAP protein repeat 3 (BIR3) domain of cIAP1have advanced into clinical trials. Here we sought to compare and contrast the biochemical activities of selected monovalent and bivalent IAP antagonists with the intent of identifying functional differences between these two classes of IAP antagonist drug candidates. The anti-cellular IAP1 (cIAP1) and pro-apoptotic activities of monovalent IAP antagonists were increased by using a single covalent bond to combine the monovalent moieties at the P4 position. In addition, regardless of drug concentration, treatment with monovalent compounds resulted in consistently higher levels of residual cIAP1 compared with that seen following bivalent compound treatment. We found that the remaining residual cIAP1 following monovalent compound treatment was predominantly tumor necrosis factor (TNF) receptor-associated factor 2 (TRAF2)-associated cIAP1. As a consequence, bivalent compounds were more effective at inhibiting TNF-induced activation of p65/NF-*κ*B compared with monovalent compounds. Moreover, extension of the linker chain at the P4 position of bivalent compounds resulted in a decreased ability to degrade TRAF2-associated cIAP1 in a manner similar to monovalent compounds. This result implied that specific bivalent IAP antagonists but not monovalent compounds were capable of inducing formation of a cIAP1 E3 ubiquitin ligase complex with the capacity to effectively degrade TRAF2-associated cIAP1. These results further suggested that only certain bivalent IAP antagonists are preferred for the targeting of TNF-dependent signaling for the treatment of cancer or infectious diseases.

## Introduction

The inhibitor of apoptosis (IAP) family of proteins is characterized by the presence of at least one baculovirus IAP protein repeat (BIR) domain. To date, eight human IAPs have been characterized.^1^ Dysregulation of the IAPs has been observed in multiple cancer cell lines and tumor samples and has been suggested as contributing to chemoresistance and treatment failure.^2–5^ The two cellular IAPs (cIAP1 and cIAP2) contain three N-terminal BIR domains, that is, BIR1, BIR2 and baculovirus IAP protein repeat 3 (BIR3), and a C-terminal really interesting new gene (RING) domain with E3 ubiquitin ligase activity.^6–8^ The distinct BIR domains of cIAP1 and cIAP2 facilitate specific and unique interactions with other proteins. For example, the BIR1 domain of cIAP1 and cIAP2 has been shown to bind tumor necrosis factor (TNF) receptor-associated factor 2 (TRAF2) during recruitment to the TNF receptor 1 (TNFR1) complex.^9–11^ The BIR2 domain of cIAP1 has been shown to interact with the N-terminus of the NF-*κ*B-inducing kinase (NIK) and promote cIAP1-dependent destabilization of NIK via ubiquitylation.^12^ Both cIAP1 and cIAP2 have been shown to homodimerize via their RING domains, which results in their auto-ubiquitylation and proteasomal degradation.^13,14^ Importantly, the BIR3 domain of cIAP1 has been shown to interact intramolecularly with the RING domain thus inhibiting intermolecular RING–RING dimerization.^13,15^ As a result, cIAP1 exists as an inactive monomer.^13^

Following TNFR1 stimulation, the receptor interacting protein kinase 1 (RIPK1) is K63 ubiquitylated by the cIAP1 E3 ligase complex which then acts as a scaffold on which the protein complex, that is, TAB2/TAB3/TAK and IKK*α*/IKK*β*/IKK*γ*, are assembled for I*κ*B phosphorylation and canonical NF-*κ*B activation.^16–20^ Loss of cIAP1 and cIAP2 leads to diminished ubiquitylation of RIPK1 and activates caspase-8.^21,22^ In mammalian cells, apoptotic cell death signaling leads to disruption of the mitochondrial outer membrane and release of the IAP antagonist proteins such as second mitochondria-derived activator of caspases (SMAC, also known as the direct IAP-binding protein with low pI (DIABLO)) and the serine protease HTRA.^23–25^ Upon release from the mitochondrial membrane, homodimeric SMAC binds to and antagonizes the anti-apoptotic function of certain IAP proteins.^23–26^ Crystallographic analysis has revealed that the N-terminal tetrapeptide of SMAC, AVPI binds to a shallow groove on the BIR3 surface of both XIAP and cIAP1.^26–29^ Both monovalent and bivalent small-molecule IAP antagonists have been designed to mimic the N-terminus of SMAC by interacting with the BIR3 domain of IAP proteins.^30,31^ These IAP antagonists promote the auto-ubiquitylation and proteasomal degradation of cIAP1 and consequently sensitize certain cancer cells to TNF-mediated apoptosis through the activation of caspase-8.^16,20,21,30–33^

In this study, we examined a selection of monovalent and bivalent IAP antagonists and found that specific bivalent IAP antagonists inhibit the TNF-mediated NF-*κ*B signal transduction pathway in cancer cells. Monovalent IAP antagonists, in contrast, were less effective at depleting TRAF2-associated cIAP1 and thus inhibiting canonical p65/NF-*κ*B activation following TNF stimulation. Owing to the pleiotropic nature of the IAPs as critical proteins not only for cell survival, but also for immunoregulation and inflammatory responses, the therapeutic utility of monovalent and bivalent IAP antagonists needs to be adjusted based on their biochemical properties.

## Results

### Bivalent IAP antagonists promoted the degradation of cIAP1 more effectively than their monovalent counterparts

**Figure 1:**
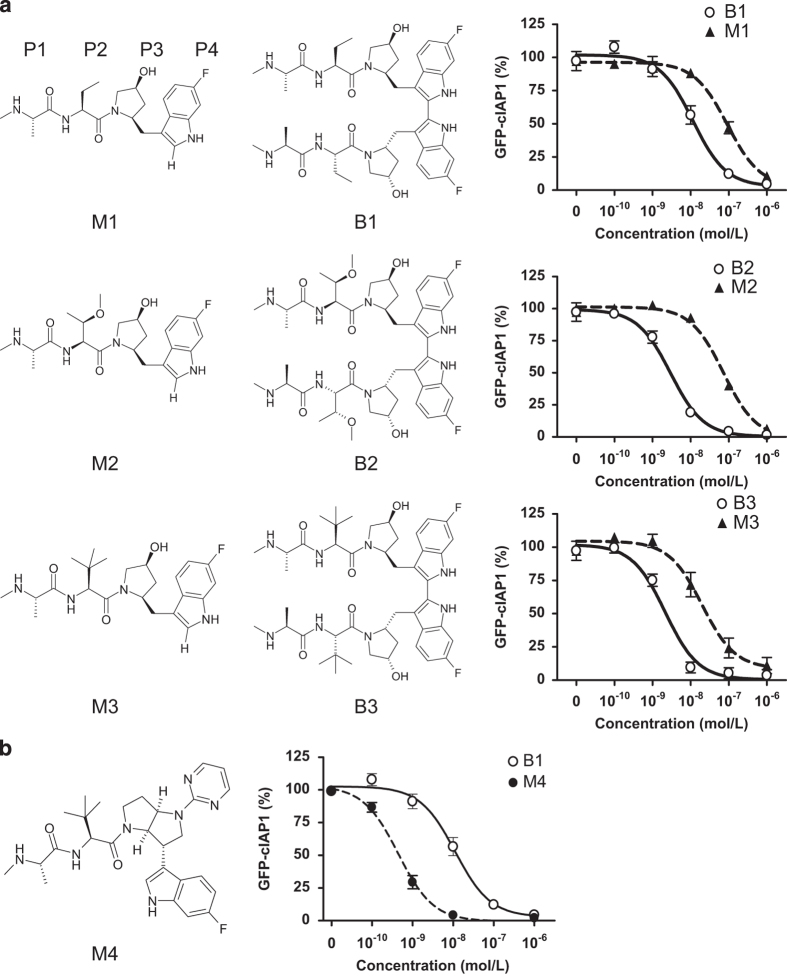
Chemical structures of selected monovalent and bivalent IAP antagonists and their ability to induce the degradation of GFP-cIAP1. (**a**) Covalent attachment at the P4 position of monovalent IAP antagonists increased their ability to induce degradation of GFP-cIAP1. P1, P2, P3 and P4: amino acid positions. (**b**) The monovalent IAP antagonist, M4, was ~20-fold more potent than B1 in the GFP-cIAP1 degradation assay. Results are mean±S.E.

In order to investigate the mechanism of cIAP1 auto-ubiquitylation and degradation induced by either monovalent or bivalent IAP antagonist treatment, we employed three pairs of bivalent and monovalent IAP antagonists (Figure 1a). The monovalent compounds comprised the individual monomeric moieties of the associated P4-linked biindole-based bivalent IAP antagonists. Recently, we demonstrated that *β*-branching of the amino acid residue at the P2 position of certain bivalent IAP antagonists was associated with pan-IAP antagonism.^34^ Monovalent IAP antagonists, M1, M2 and M3, possessed aminobutyric acid (Abu), *O*-methyl-threonine [Thr(Me)] or *tert*-leucine (Tle), at the P2 position, respectively, which corresponded to the respective bivalent IAP antagonists B1, B2 and B3. Birinapant (B1) is currently in clinical trials for the treatment of solid tumors, hematological malignancies and hepatitis B virus (HBV) infection.^35–37^ The binding affinity for these monovalent and bivalent IAP antagonists to the isolated cIAP1 BIR3 domain was comparable as determined using a fluorescence polarization (FP) assay (Table 1).^32^ We have previously shown that IAP antagonist-induced degradation of green fluorescent protein (GFP)-tagged-cIAP1 faithfully recapitulated the degradation of endogenous cIAP1 in A375 cells.^32^ As shown in Figure 1a and Table 1, bivalent IAP antagonists B1, B2 and B3 were ~10-fold more potent in the GFP-cIAP1 degradation assay than M1, M2 and M3, respectively.

### Monovalent IAP antagonists failed to induce the quantitative loss of cIAP1

**Figure 2:**
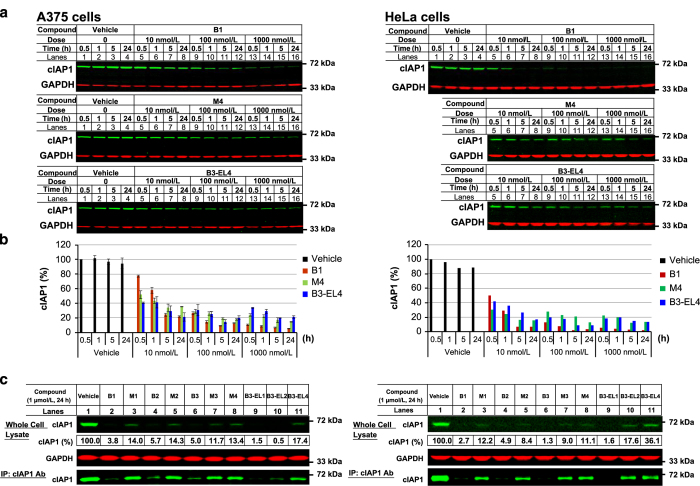
Monovalent IAP antagonists and B3-EL4 showed reduced ability to degrade cIAP1 compared with B1. (**a**) A375 and HeLa cells were treated with various concentrations of B1, M4 or B3-EL4 for the time indicated. Western blots of A375 and HeLa cells are representative of three and two independent experiments, respectively. (**b**) The intensity of cIAP1 detected by western blot analysis shown was quantified by normalization with the intensity of GAPDH using Odyssey infrared imaging system (LI-COR Biosciences Inc., Lincoln, NE, USA). The results of A375 cells are mean±S.D. from two replicates of a single experiment: HeLa results are from a single experiment. (**c**) Residual cIAP1 levels in whole-cell lysates were evaluated using mouse monoclonal and goat polyclonal anti-cIAP1 antibodies for IP and western blot analysis, respectively. The data shown are representative of two independent experiments. The intensity of cIAP1 detected in the whole-cell lysate samples was quantified by normalization with the intensity of GAPDH using Odyssey infrared imaging system.

For comparative purposes, we developed a structurally unique monovalent compound, M4, containing Tle at the P2 position, which was ~100-fold more potent than M1, M2 or M3 in the GFP-cIAP1 degradation assay (Table 1). Relative to the clinical compound B1, M4 was ~20-fold more potent in the GFP-cIAP1 assay and thus, in this analysis, M4 represented the most potent monovalent IAP antagonist in our biochemical assays (Figure 1b and Table 1). Consistent with these results, M4 and B1 were effective at decreasing endogenous cIAP1 levels when assessed by western blot analysis (Figures 2a and b). Although M4 appeared to induce a more rapid loss of cIAP1 than B1 at ⩽1 h post-IAP antagonist addition (Figures 2a and b), B1 reduced cIAP1 to lower levels than M4 upon longer incubation or at higher concentrations (Figure 2b). Similar results were observed in both A375 and HeLa cells treated with the same IAP antagonists (Figures 2a and b).

In an attempt to further document the differences between bivalent and monovalent compounds, immunoprecipitation (IP) experiments using an anti-cIAP1 antibody were performed. In agreement with the results shown in Figures 2a and b, IP experiments demonstrated that B1 treatment depleted >95% of the total cIAP1 protein (Figure 2c, lanes 1 and 2). In contrast, ~20% cIAP1 remained following M4 treatment (Figure 2c, lane 8). The bivalent compounds, B2 and B3, showed similar results to B1 (Figure 2c, lanes 4 and 6). Treatment with each monovalent compound resulted in a higher residual cIAP1 level when compared with the bivalent IAP antagonists (Figure 2c lanes 3, 5 and 7). Although these differences in residual cIAP1 levels following monovalent or bivalent IAP antagonist treatment were modest, they were readily demonstrated by immunoprecipitating cIAP1 and the differences were observed in multiple experiments in both cell lines. These results suggested that a portion of cIAP1 existed which was resistant to monovalent IAP antagonist treatment despite comparable binding affinities to the cIAP1 BIR3 domain, and induction of cIAP1 auto-ubiquitylation and degradation.

### Increasing linker length of bivalent IAP antagonists negatively impacted cIAP1 loss

By virtue of intramolecular cIAP1 RING–BIR3 interactions, monomeric cIAP1 exists in an auto-inhibited state.^13,15^ However, cIAP1 requires intermolecular RING dimerization to function as an E3 ubiquitin ligase enzyme. We have previously demonstrated that bivalent IAP antagonists such as B1, but not monovalent IAP antagonists, stabilized the cIAP1 RING dimer possibly by simultaneous occupation of both cIAP1 BIR3 domains, that is, by BIR3 domain cross-linking.^13,34^ We next addressed whether the linker length between the two monomer halves of a bivalent IAP antagonist would impact cIAP1 auto-degradation.

Two series of bivalent IAP antagonists that separated the monomer halves of B1 or B3 by 1 (extended linker=methylene, EL1), 2 (extended linker=ethylene, EL2) or 4 (extended linker=butylene, EL4) carbon atoms were prepared (thus, ‘bivalent-extended linker’, B-EL); each series independently maintained Abu or Tle at the respective P2 positions (Supplementary Figure S1). Because these structurally related analogs differed only in the length of the linker chain, the *in vitro* binding affinities to the isolated cIAP1 BIR3 domain were unchanged, as determined by the FP assay (Table 1). In the GFP-cIAP1 degradation assay, the linker-extended B1 analogs (P2=Abu) lost activity in a linker length-dependent fashion, that is, B1>B1-EL1>B1-EL2≈B1-EL4, suggesting that unlike B1, these linker-extended analogs were less able to stabilize the cIAP1 E3 ligase complex.

In contrast, when P2=Tle, B3 and each of the linker-extended analogs, that is, B3-EL1, B3-EL2 and B3-EL4, maintained a comparable ability to degrade GFP-cIAP1, likely due to the increased hydrophobicity of the Tle residue relative to Abu (Supplementary Figure S1). Strikingly, however, despite the low IC_50_ value in the GFP-cIAP1 assay, treatment of A375 and HeLa cells with B3-EL4 resulted in higher levels of residual cIAP1 than either B1 or B3 treatment (Table 1 and Figure 2). These results were similar to those observed following treatment with M4 or other monovalent IAP antagonists (Figure 2), suggesting that a fraction of cIAP1 remained resistant to a subset of IAP antagonists.

### Both monovalent and bivalent IAP antagonists promoted RIPK1:caspase-8 complex formation and apoptosis in sensitive cancer cell lines

**Figure 3:**
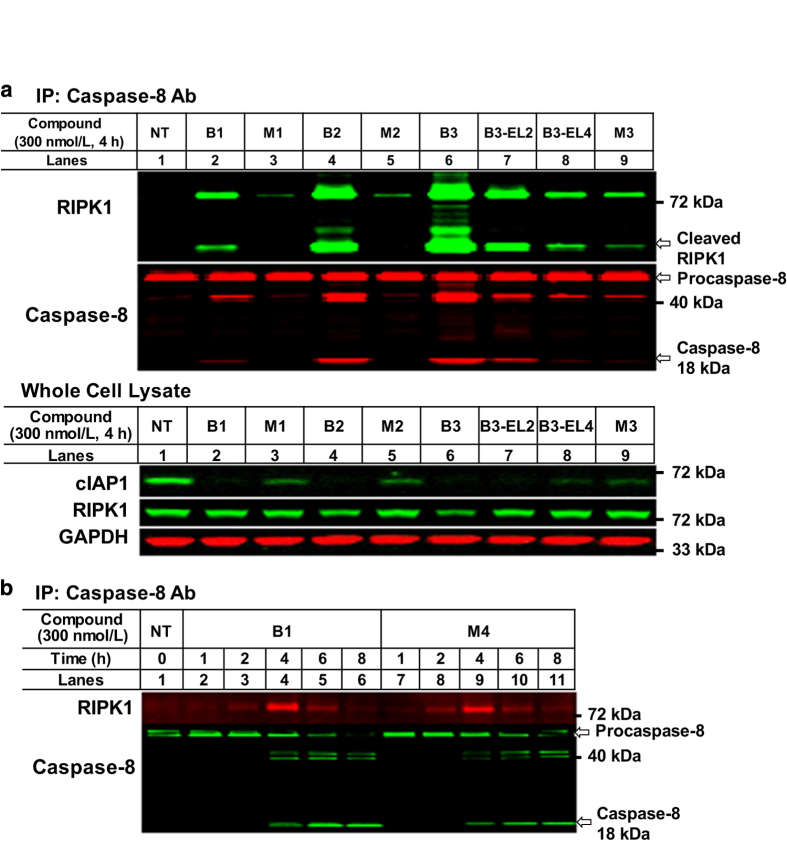
Both monovalent and bivalent IAP antagonists promoted RIPK1:caspase-8 complex formation. (**a**) RIPK1:caspase-8 complex formation by IAP antagonist treatment of EVSA-T cells. Following IAP antagonist treatment, the whole-cell lysate was incubated with anti-caspase-8 antibody and the RIPK1:caspase-8 complex was evaluated by western blot analysis using anti-RIPK1 antibody. Representative result from two independent experiments. (**b**) Comparison between B1 and M4 treatment in RIPK1:caspase-8 complex formation and subsequent activation of caspase-8 in EVSA-T cells. B1 and M4 were comparable in inducing the RIPK1:caspase-8 complex and caspase-8 activation in a time-dependent manner. Representative result from two independent experiments. Note: B1 and M4 showed similar cytotoxicity in EVSA-T cells (Table 1).

In IAP antagonist-sensitive cancer cells, depletion of cIAP1 following IAP antagonist treatment resulted in the formation of a RIPK1:caspase-8 complex with subsequent activation of caspase-8.^16,32,38^ To address the fraction of cIAP1 that remained following monovalent IAP antagonist treatment, we first considered the induction of the RIPK1:caspase-8 complex by monovalent or bivalent IAP antagonist treatment in EVSA-T cells, an IAP antagonist-sensitive breast cancer cell line. Following IAP antagonist treatment, EVSA-T-cell lysates were subjected to IP using anti-caspase-8 antibody, then immunoblotted with the anti-RIPK1 antibody. As shown in Figure 3, both monovalent and bivalent antagonists promoted RIPK1:caspase-8 complex formation albeit to varying degrees. Bivalent IAP antagonists, B1, B2 and B3, more efficiently induced the RIPK1:caspase-8 complex (Figure 3a, lanes 2, 4 and 6) compared with the corresponding monovalent analogs, that is, M1, M2 and M3 (Figure 3a, lanes 3, 5 and 9, respectively). In a linker-dependent fashion, treatment with B3-EL2 or B3-EL4 resulted in reduced formation of the RIPK1:caspase-8 complex, which was consistent with their reduced capacity to induce cell death in the EVSA-T-cell line (Figure 3a, lanes 6–8 and Table 1). In addition, treatment with either M4- or B1- induced RIPK1:caspase-8 complex formation and activated caspase-8 which correlated with their abilities to induce EVSA-T-cell death (Figure 3b and Table 1). These results suggested that cIAP1 degradation was necessary for the formation of the RIPK1:caspase-8 complex and that RIPK1:caspase-8 complex formation was associated with cytotoxicity in EVSA-T cells. Consistent with this data, similar results were observed in the IAP antagonist-sensitive MDA-MB-231 triple-negative breast cancer cell line (Supplementary Figure S2). Thus, under these experimental conditions, both monovalent and bivalent IAP antagonist treatment resulted in sufficient cIAP1 loss to support RIPK1:caspase-8 complex formation and induction of apoptosis in sensitive cancer cell lines.

### Bivalent IAP antagonists, but not monovalent IAP antagonists, depleted cIAP1 from TRAF2

**Figure 4:**
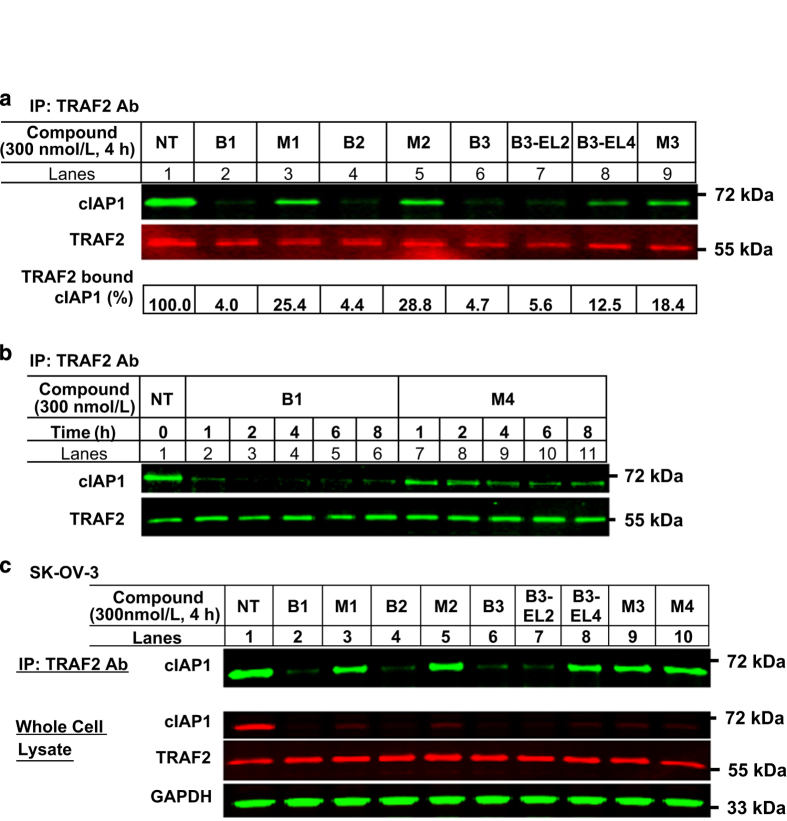
Monovalent IAP antagonists and linker chain-extended bivalent analog B3-EL4 were less efficient at inducing the degradation of TRAF2-associated cIAP1. (**a**) The whole-cell lysates of EVSA-T cells prepared in Figure 3 were subjected to co-IP using anti-TRAF2 antibody. The IP complex was evaluated by the western blot analysis with anti-cIAP1 antibody. Representative result from two independent experiments. (**b**) Comparison between B1 and M4 in degrading TRAF2-associated cIAP1 in EVSA-T cells. M4 treatment resulted in higher residual levels of TRAF2-associated cIAP1 than B1 treatment throughout the time points investigated. Representative result from two independent experiments. (**c**) SK-OV-3 cells were incubated in the presence or absence of 300 nmol/l of IAP antagonist for 4 h. Whole-cell lysates were subjected to IP using anti-TRAF2 antibody. The IP complex was evaluated by the western blot analysis with anti-cIAP1 antibody. Representative result from two independent experiments.

We next sought to characterize the pool of cIAP1 that remained after monovalent IAP antagonist treatment of EVSA-T cells. We have previously shown that TRAF2-associated cIAP1 comprised ~25% of the total cIAP1 expressed in HeLa cells and that B1 treatment was capable of degrading both TRAF2- and non-TRAF2-associated cIAP1 and cIAP2 in this cell line.^32^ We hypothesized that the cIAP1 remaining after monovalent IAP antagonist treatment of EVSA-T cells might be partly attributable to residual TRAF2-associated cIAP1. To address this hypothesis, whole-cell lysates of EVSA-T cells, which were prepared in the experiment described in Figure 3, were subjected to co-IP using an anti-TRAF2 antibody (Figure 4). Quantitative assessment of the intensities of total and TRAF2-associated cIAP1 revealed that ~30% of cIAP1 was associated with TRAF2 in EVSA-T cells (total cIAP1 in whole-cell lysate is shown in Figure 3a, bottom panel, lane 1 and TRAF2-associated cIAP1 is shown in Figure 4a, lane 1). Treatment with bivalent IAP antagonists, B1, B2 and B3, depleted >95% of TRAF2-associated cIAP1 in EVSA-T cells (Figure 4a, lanes 2, 4 and 6). In contrast, ~20–25% of the TRAF2-associated cIAP1 remained after treatment with monovalent IAP antagonists including M4 (Figure 4a, lanes 3, 5 and 9 and Figure 4b). Notably, TRAF2-associated cIAP1 was resistant to M4 treatment throughout the time points investigated (Figure 4b). Similar results were also obtained using SK-OV-3 cells (Figure 4c). These results suggested that bivalent IAP antagonists, but not monovalent antagonists, induced formation of an active cIAP1 E3-ligase complex that could efficiently deplete TRAF2-associated cIAP1. Furthermore, while B3-EL2-depleted TRAF2-associated cIAP1 to levels comparable to B3 and other bivalent IAP antagonists, B3-EL4 failed to deplete TRAF2-associated cIAP1 to the same extent (Figure 4a, lanes 7 and 8). This result implied that B3-EL4 interacted with cIAP1 in a manner akin to monovalent IAP antagonists.

### Bivalent IAP antagonists are more effective than monovalent IAP antagonists at inhibiting the TNF-mediated NF-*κ*B signal transduction pathway

**Figure 5:**
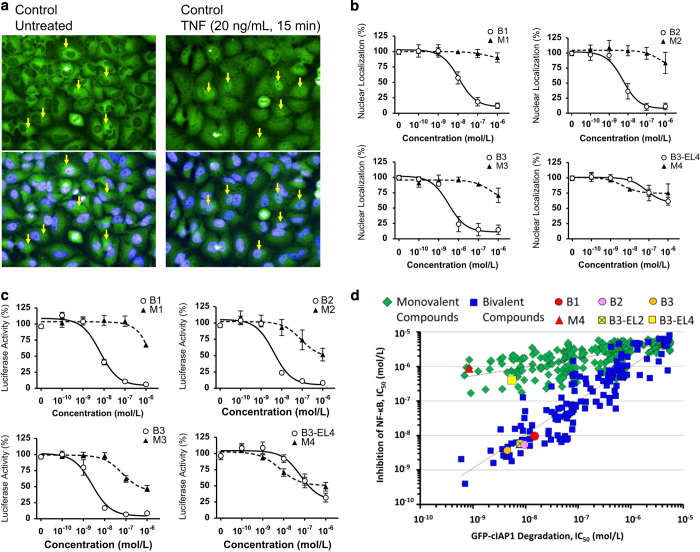
Bivalent IAP antagonists inhibited the nuclear translocation and the transcriptional activity of p65/NF-*κ*B following TNF stimulation. (**a**) The nuclear translocation of p65/NF-*κ*B in HeLa cells was evaluated using the Operetta High Content Imaging System (PerkinElmer, Waltham, MA, USA), as described in Materials and Methods section. The nuclear DNA was counter-stained with Hoechst 33342: the blue nuclear stain is shown in each of the bottom panels. Arrows indicate p65/NF-*κ*B localized in cytoplasm (perinuclear) or nuclei in unstimulated (left panel) and TNF-stimulated (right panel) HeLa cells, respectively. Note the fluorescence intensity of p65/NF-*κ*B increased in some of the cells stimulated with TNF, whereas the perinuclear p65/NF-*κ*B intensity was reduced following TNF stimulation. (**b**) Bivalent IAP antagonist showed efficient inhibition of the nuclear translocation of p65/NF-*κ*B in HeLa cells. Quantitative evaluation of p65/NF-*κ*B in the nuclear or cytoplasm in HeLa cells using Operetta High Content Imaging System. Cells were treated with IAP antagonist for 2 h, followed by TNF stimulation. Representative result from three independent experiments (mean±S.E.). (**c**) Bivalent IAP antagonist showed efficient inhibition of the transcriptional activity in the p65/NF-*κ*B reporter gene assay. The transcriptional activity of p65/NF-*κ*B was evaluated using HeLa cells stably harboring p65/NF-*κ*B reporter gene. Results are mean±S.E. (**d**) Despite efficient degradation of GFP-cIAP1, monovalent IAP antagonists and B3-EL4 were less effective at inhibiting the activation of p65/NF-*κ*B following TNF stimulation in comparison with bivalent IAP antagonists.

The TRAF2–cIAP1 protein complex is an essential component in promoting the nuclear translocation of p65/NF-*κ*B and activation of the canonical NF-*κ*B signal transduction pathway following TNF stimulation.^39,40^ We next addressed the effects of IAP antagonist treatment on TNF-mediated NF-*κ*B signaling in HeLa cells (Figure 5). An immunofluorescence study using anti-p65/NF-*κ*B antibody revealed that p65/NF-*κ*B translocated into the nucleus in response to TNF stimulation in HeLa cells (Figure 5a) and bivalent IAP antagonists, B1, B2 and B3, inhibited the nuclear translocation of p65/NF-*κ*B induced by TNF in a dose-dependent manner (Figure 5b and Supplementary Figure S3). In agreement with this observation, B1, B2 and B3 inhibited the transcriptional activity of p65/NF-*κ*B, as determined by an NF-*κ*B luciferase reporter gene assay (Figure 5c). The activity of B1 toward inhibition of p65/NF-*κ*B-dependent gene transcription in HeLa cells was comparable to Sulfasalazine, an anti-inflammatory agent used in the management of inflammatory bowel disease and rheumatoid arthritis (Supplementary Figure S4). In contrast, neither B3-EL4 nor monovalent IAP antagonists inhibited the nuclear translocation of p65/NF-*κ*B or expression of luciferase following TNF stimulation under these experimental conditions (Figures 5a–c and Supplementary Figure S3). We further investigated the correlation between GFP-cIAP1 degradation and inhibition of p65/NF-*κ*B-mediated luciferase activity with >300 monovalent and bivalent IAP antagonists, including previously reported monovalent and bivalent IAP antagonists,^16,41–44^ whose IC_50_ values from either assay ranged over three logarithmic orders. As shown in Figure 5d, bivalent and monovalent IAP antagonists revealed different correlation patterns (*P*<0.001) due to only partial inhibition of TNF-mediated p65/NF-*κ*B activation by monovalent IAP antagonists including the potent monovalent ligand, M4. These results suggested that the residual TRAF2-associated cIAP1 following monovalent IAP antagonist treatment was sufficient to sustain the TNF-mediated NF-*κ*B signaling pathway. Furthermore, these results demonstrated that bivalent and monovalent IAP antagonists could be distinguished biochemically by their abilities to induce the degradation of TRAF2-associated cIAP1 and inhibit TNF-mediated p65/NF-*κ*B signal transduction.

### Inhibition of nuclear translocation of p65/NF-*κ*B by bivalent IAP antagonist is cell type-dependent

**Figure 6:**
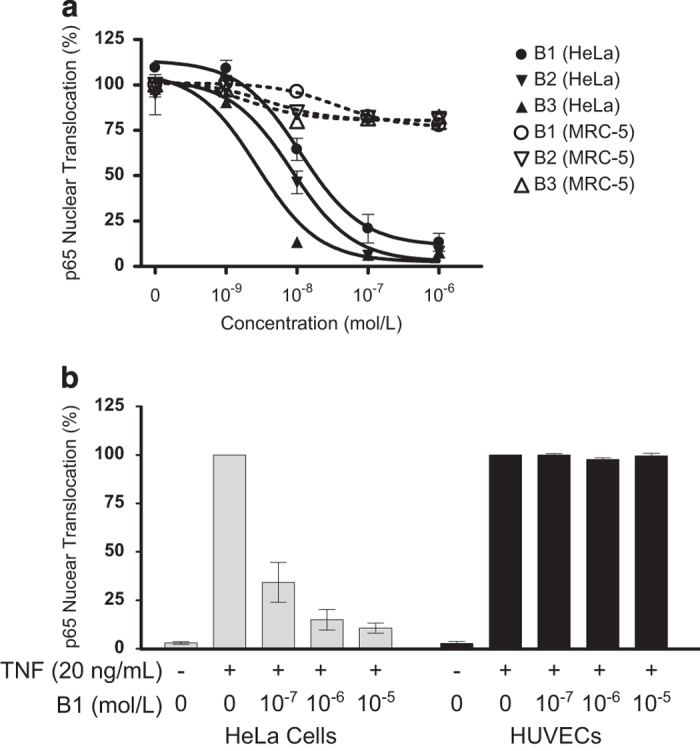
Bivalent IAP antagonists did not inhibit the nuclear translocation of p65/NF-*κ*B following TNF stimulation in MRC-5 fibroblast or HUVECs. (**a** and **b**) Quantitative evaluation of p65/NF-*κ*B in the nucleus or cytoplasm in MRC-5 (**a**) and HUVECs (**b**) using HeLa cells as controls. Nuclear translocation was evaluated using the Operetta High Content Imaging System as illustrated in Figure 5. Representative result from three (**a**) and two (**b**) independent experiments.

The inhibition of TNF-mediated nuclear translocation of p65/NF-*κ*B by bivalent IAP antagonists was further assessed in non-cancerous human umbilical vein endothelial cells (HUVECs) and MRC-5 fibroblast cells. Treatment with B1, B2 or B3 resulted in cIAP1 loss in MRC-5 cells (data not shown), but unlike HeLa cells, this did not result in the inhibition of TNF-mediated p65/NF-*κ*B nuclear translocation (Figure 6a). Similarly, in comparison with HeLa cells, B1 treatment of HUVECs showed no inhibition of p65/NF-*κ*B nuclear translocation following TNF stimulation (Figure 6b). These results suggested that the effect of these bivalent IAP antagonists on p65/NF-*κ*B nuclear translocation is cell type-dependent.

## Discussion

cIAP1 and cIAP2 are single-subunit RING-containing E3 ubiquitin ligases and are recruited to TNFR complexes in order to modulate the receptor-mediated pathways leading to canonical p65/NF-*κ*B activation.^7,8,10^ Ubiquitin transfer, or ubiquitylation, is a highly orchestrated process involving multiple proteins including a substrate protein, an E2–Ub conjugate and an E3 ubiquitin ligase, that is, RING-dimerized cIAP1 in this instance. IAP antagonists bind to the cIAP1 BIR3 domain and induce a conformational rearrangement, which promotes rapid RING domain-mediated dimerization, auto-ubiquitylation and cIAP1 degradation via the ubiquitin–proteasome system.^13,45^ The IAPs function as molecular switches between pro-survival and pro-apoptotic pathways at the TNFR1 and the loss of cIAP1 by IAP antagonist treatment results in formation of a RIPK1:caspase-8 complex and activation of the extrinsic apoptotic pathway in a subset of cancer cell lines.

In this study, we compared monovalent and bivalent IAP antagonists in the context of cIAP1 auto-ubiquitylation/degradation in sensitive cancer cells and found fundamental differences between these classes of IAP antagonists. We observed that a series of indole-based monovalent IAP antagonists were more effective inducers of cIAP1 loss when dimerized at the P4 position via a single covalent bond. We also showed that both monovalent and bivalent IAP antagonists including linker-extended compounds induced cIAP1 depletion and promoted RIPK1:caspase-8 complex formation, which correlated with their ability to degrade GFP-cIAP1 and induce cytotoxicity in cancer cell lines. In contrast, monovalent and bivalent IAP antagonists showed differences in the degradation of cIAP1, particularly at the TRAF2 complex. Bivalent IAP antagonists, B1, B2 and B3, induced the quantitative degradation of both TRAF2- and non-TRAF2-associated cIAP1, whereas monovalent IAP antagonists were less effective against TRAF2-associated cIAP1. In addition, the quantitative loss of TRAF2-associated cIAP1 following bivalent IAP antagonist treatment correlated with inhibition of TNF-stimulated p65/NF-*κ*B nuclear translocation and gene transcription in the cancer cell lines tested. The inhibition of p65/NF-*κ*B pathway following bivalent IAP antagonist treatment of sensitive cancer cells may offer an explanation as to why bivalent IAP antagonists are more cytotoxic than the structurally related monovalent IAP antagonists. Intriguingly, bivalent IAP antagonists did not inhibit the TNF-mediated p65/NF-*κ*B nuclear translocation in MRC-5 fibroblast cells or HUVECs despite IAP antagonist-mediated degradation of cIAP1, suggesting that cIAP1 loss is required but not sufficient to inhibit TNF-mediated p65/NF-*κ*B nuclear translocation in certain cell lines.

Although we cannot rule out the involvement of other mechanisms for the efficient inhibition of p65/NF-*κ*B following bivalent IAP antagonist treatment, we further demonstrated that the IAP antagonist-induced loss of TRAF2-associated cIAP1 and subsequent inhibition of TNF-mediated p65/NF-*κ*B signaling was negatively impacted by extension of the linker chain between the two monovalent IAP antagonist moieties. The loss of activity by linker chain extension was partly overcome by incorporation of the Tle residue at the P2 position; likely due to increased hydrophobicity near the central portion of the IAP antagonist, resulting in reduced diffusion away from the cIAP1 BIR3 binding groove. However, at the longest chain length tested, B3-EL4 treatment resulted in only partial TRAF2-associated cIAP1 loss and failed to inhibit the TNF-induced p65/NF-*κ*B pathway. This result confirmed that bivalent IAP antagonists linked at P4 by only a single covalent bond degraded both TRAF2-associated and non-TRAF2-associated cIAP1 and inhibited TNF-mediated p65/NF-*κ*B activation, whereas linker-extended variants like B3-EL4 displayed biochemical characteristics of a monovalent IAP antagonist. The inability of B3-EL4 or monovalent IAP antagonists to induce quantitative cIAP1 loss might reflect the instability of these IAP antagonist-induced cIAP1 E3 complexes. Alternatively, monovalent IAP antagonists might be less able to form the TRAF2-associated protein complex at reduced cIAP1 levels. A more in-depth biophysical analysis of the cIAP1 auto-ubiquitylation process will be required to address these fundamental mechanistic questions.

Monovalent and bivalent IAP antagonists have been demonstrated to induce cancer cell death *in vitro* and *in vivo* and several compounds are in development for the treatment of cancer.^15,30^ Lalaoui *et al.*^46^ recently demonstrated that birinapant (B1) treatment with the p38 inhibitor, LY2228820, was well-tolerated and increased cancer cell death in a highly aggressive oncogenic NRas mutant model of AML. Other studies have shown that IAPs are critical regulators of multiple pathways that include both innate and adaptive immune cell functions.^47–50^ TNF is an important pro-inflammatory cytokine involved in mediating cell death and inflammation in many human diseases such as rheumatoid arthritis, viral infection and cancer. Thus, although the activity of bivalent IAP antagonists toward inhibiting p65/NF-*κ*B may vary depending on cell type *in vivo*, IAP antagonists might offer therapeutic opportunities for diseases associated with inflammation and immune regulation in addition to their anti-tumor properties. We have shown that mice carrying liver-specific cIAP1/2 deficiency exhibited enhanced clearance of HBV infection compared with wild-type animals.^36^ In addition, using an immunocompetent C57BL/6 mouse model of chronic HBV infection, treatment with bivalent IAP antagonists including birinapant (B1), but not a monovalent antagonist, resulted in effective clearance of HBV in a TNF-dependent manner.^37^ The ability to reduce serum HBV DNA by bivalent IAP antagonist treatment may be reflective of the differing biological properties between monovalent and bivalent IAP antagonists in relation to TNF-dependent signaling.

It has been shown that loss of cIAP1 following monovalent IAP antagonist treatment, that is, LBW-242, was TRAF2-dependent and that the LBW-242-induced loss of cIAP2 was both TRAF2- and cIAP1-dependent.^51^ In contrast, bivalent IAP antagonists were able to induce degradation of GFP-cIAP2 expressed in cIAP1/cIAP2 double knockout mouse embryonic fibroblast cells suggesting a cIAP1-independent role for cIAP2 degradation (data not shown). The results presented in this study provide new insights into the use of IAP antagonists by further characterizing IAP-dependent biochemical activities which now include cIAP1 degradation via ubiquitylation, RIPK1-dependent caspase-8 activation, TNF-dependent p65/NF-*κ*B signaling and caspase-3/7 or -9 repression.^50^ And, that these activities might be selectively exploited by employing either monovalent or bivalent IAP antagonists. Future clinical applications of monovalent or bivalent IAP antagonists, therefore, should be tailored based on the required IAP antagonism profile and the biochemical properties of these distinct classes of pro-apoptotic ligands.

## Materials and Methods

### Cell culture

SK-OV-3 and MDA-MB-231 cells were obtained from the American type culture collection (ATCC, Manassas, VA, USA). EVSA-T cells were obtained from the Leibniz-Institut DSMZ-Deutsche Sammlung von Mikroorganismen und Zellkulturen GmbH (Braunschweig, Germany). HeLa cells were obtained from the European Collection of Cell Cultures (Sigma-Aldrich Co. LLC, St Louis, MO, USA). A375 cells were obtained from Pharmaceutical Product Development, Inc. (Wayne, PA, USA). All cell lines have not been authenticated by TetraLogic Pharmaceuticals (Malvern, PA, USA). According to the recommendations by the providers, all cell lines were maintained in appropriate medium containing 10% heat-inactivated fetal bovine serum from HyClone (Logan, UT, USA) and were grown in a humidified, 37 °C, 5% CO_2_ incubator.

### Reagents

Hoechst 33342, Protein A/G agarose and a NuPAGE system were purchased from Invitrogen Life Technologies (Grand Island, NY, USA). AbuRPFK(5-Fam)-NH_2_ peptide was obtained from Biomer Technologies (Pleasanton, CA, USA). Anti-cIAP1 antibody for western blot analysis was purchased from R&D Systems (Minneapolis, MN, USA). Anti-GAPDH, anti-caspase-8 and anti-PARP antibodies were obtained from Santa Cruz Biotechnology (Dallas, TX, USA). Anti-RIPK1 antibody was obtained from BD Biosciences (Franklin Lakes, NJ, USA). Anti-TRAF2 antibody was obtained from Cell Signaling (Danvers, MA, USA). IAP antagonists were synthesized at TetraLogic Pharmaceuticals.

### Co-immunoprecipitation and western blot analysis

For immunoprecipitation studies, cells were collected and lysed with cell lysis buffer consisting of 20 mmol/l Tris-HCl (pH 7.5), 150 mmol/l sodium chloride, 10% glycerol, 2 mmol/l EDTA, 50 mmol/l sodium fluoride, 25 mmol/l *β*-glycerophosphate, 0.2 mmol/l sodium vanadate, 10 mmol/l sodium pyrophosphate, 0.5% Triton X-100, 1 mmol/l phenylmethylsulfonyl fluoride and protease inhibitor cocktail (Roche/Sigma-Aldrich Co. LLC). Crude cell lysate was cleared by centrifugation with 14000 r.p.m. for 10 min at 4 °C. The cleared whole-cell lysate (2–5 mg) was incubated with an antibody against cIAP1 (Santa Cruz Biotechnology), TRAF2 (Cell Signaling) or caspase-8 (Santa Cruz Biotechnology) as indicated in the figure, overnight at 4 °C. The immuno-protein complex was purified using Protein A/G agarose beads and subjected to western blot analysis. Protein samples were applied to NuPAGE gel, followed by electrotransfer to Immobilon-FL membranes (Millipore, Billerica, MA, USA). Membranes were incubated in blocking buffer (LI-COR Bioscience Inc., Lincoln, NE, USA). Detection of proteins on the membrane was performed with a standard procedure using selected primary antibodies and an appropriate secondary antibody conjugated to Alexa Fluor 680 or 800 IRDye. Membranes were scanned and analyzed by Odyssey infrared imaging system (LI-COR Bioscience, Inc.).

### Fluorescence polarization assay

The binding affinities of compounds to the purified BIR3 domain of cIAP1 was determined as described previously and are reported as inhibitory constant (Ki) values.^32,52^

### Green fluorescent protein–cIAP1 degradation assay

The ability of monovalent and bivalent IAP antagonists to induce cIAP1 degradation was evaluated using A375 melanoma cells stably expressing GFP-fusion cIAP1, HA2×EGFP-cIAP1, as previously described.^32^ The inhibitory concentration for 50% GFP intensity (IC_50_) was derived by non-linear regression analysis using GraphPad Prism 4.0 (La Jolla, CA, USA).

### Cell viability assay

Viability was determined using the MTT [3-(4,5-dimethylthiazol-2-yl)-2,5-diphenyl tetrazolium bromide] assay with 96-well plates containing 10 000 cells per well. After the cells were allowed to adhere overnight, serial dilutions of IAP antagonists were added and plates were incubated for 72 h. MTT was added to a final concentration of 1 mg/ml and cell viability was measured following the manufacturer’s recommendation. The cytotoxic concentration for 50% cell death (CC_50_) was derived by non-linear regression analysis using GraphPad Prism 4.0.

### p65/NF-*κ*B nuclear translocation analysis

The nuclear translocation of p65/NF-*κ*B in HeLa cells was evaluated by immunofluorescence analysis using Alexa Fluor-conjugated anti-p65/NF-*κ*B antibody (sc-8008AF488, Santa Cruz Biotechnology). HeLa cells, cultured in black wall 96-well plates, were treated with IAP antagonists at 37 °C for 2 h, followed by TNF (20 ng/ml) stimulation for the time indicated. Cells were then fixed with 4% paraformaldehyde (pH 7.2) for 10 min at ambient temperature. Following washing with phosphate-buffered saline (PBS) three times, the cells were incubated with Alexa Fluor-conjugated anti-p65/NF-*κ*B antibody suspended in KB buffer (10 mmol/l Tris-HCl, pH 7.5, 150 mmol/l NaCl, 0.1% BSA) at 4 °C overnight. Hoechst 33342 was added to counter-stain the nuclear DNA for 5 min and the cells were then washed three times with PBS. Fluorescence intensities of p65/NF-*κ*B and Hoechst 33342 were quantitatively evaluated from ~1200 cells in each treatment condition by using Operetta High Content Imaging System (PerkinElmer, Waltham, MA, USA).

### p65/NF-*κ*B reporter gene assay

HeLa cells that carry an NF-*κ*B luciferase reporter gene were utilized for quantitative measurement of p65/NF-*κ*B activity following TNF stimulation. The cells seeded in 96-well plates were treated with various concentrations of IAP antagonist 2 h prior to the TNF stimulation for 4 h. The luciferase activity was measured by using Steady-Glo Luciferase Assay System (Promega Corp., Madison, WI, USA).

## Figures and Tables

**Table 1:** Biochemical activities of IAP antagonist employed in this study

*Compound*	*cIAP1 BIR3 binding (Ki, nmol/l)*	*GFP-cIAP1 degradation (2 h, IC*_*50*_*±S.D., nmol/l)*	*GFP-cIAP2 degradation (2 h, IC*_*50*_*±S.D., nmol/l)*	*Cytotoxicity/MTT (72 h, CC*_*50*_*±S.D., nmol/l)*		
				*SK-OV-3*	*EVSA-T*	*MDA-MB-231*
M1	~1	118±52	807±33	66±30	165±22	604±168
M2	~1	74±18	715±111	18±13	95±28	550±221
M3	~1	46±27	266±118	4±1	21±1	221±99
M4	~1	0.8±0.1	31±9	0.05±0.02	2.0±1.6	22±17
B1	~1	15±4	109±52	1.1±0.9	1.6±0.2	6±3
B2	~1	5±2	30±10	1.0±0.5	0.2±0.1	1.2±0.7
B3	~1	4±2	6±2	0.01± 0.002	0.1±0.02	0.2±0.1
B1-EL1	~1	74±13	1294±479	8±6	8±3	73±33
B1-EL2	~1	240±112	1000±201	30±27	23±21	169±83
B1-EL4	~1	297±86	3730±450	279±121	32±31	379±106
B3-EL1	~1	9±2	50±7	0.4±0.1	0.2±0.1	0.8±0.4
B3-EL2	~1	8±2	62±14	0.2±0.04	0.4±0.3	2.1±1.5
B3-EL4	~1	8±1	55±14	220±72	8±5	50±21

## Abbreviations

Ab: antibody
Abu: aminobutyric acid
AVPI: alanine-valine-proline-isoleucine tetrapeptide
BIR: baculovirus IAP repeat domain
cIAP1: cellular inhibitor of apoptosis protein 1
DIABLO: direct IAP-binding protein with low pI (also, SMAC)
EL: extended linker
GAPDH: glyceraldehyde 3-phosphate dehydrogenase
GFP: green fluorescent protein
HTRA: high-temperature requirement A protease
HUVECs: human umbilical vein endothelial cells
IAP: Inhibitor of apoptosis proteins
IKKα/IKKβ/IKKγ: inhibitor of κB kinase alpha/ inhibitor of κB kinase beta/ inhibitor of κB kinase gamma
kDa: kilodalton
MTT: 3-(4,5-dimethylthiazol-2-yl)-2,5-diphenyltetrazolium bromide
NF-κB: nuclear factor kappa-light-chain-enhancer of activated B cells
NIK: NF-κB-inducing kinase
NT: no treatment
RING: really interesting new gene domain
RIPK1: receptor interacting protein kinase 1
SMAC: second mitochondria-derived activator of caspases (also, DIABLO)
TAB2/TAB3: TAK1-binding proteins 2 and 3
TAK1: TGFβ-activated kinase 1
Thr(Me): threonine *O*-methyl ether
Tle: *tert*-leucine
TNF: tumor necrosis factor
TNFR1: TNF receptor 1
TRAF2: TNF receptor-associated factor 2
Ub: ubiquitin
XIAP: X chromosome-linked IAP
