# Supplementary figures and images for: Bivalent IAP antagonists, but not monovalent IAP antagonists, inhibit TNF-mediated NF-κB signaling by degrading TRAF2-associated cIAP1 in cancer cells
Source: Cell Death Discov. 2017 Jan 16;3:16046–. doi: 10.1038/cddiscovery.2016.46 (PMC5238498; doi:10.1038/cddiscovery.2016.46)

## Slide 1
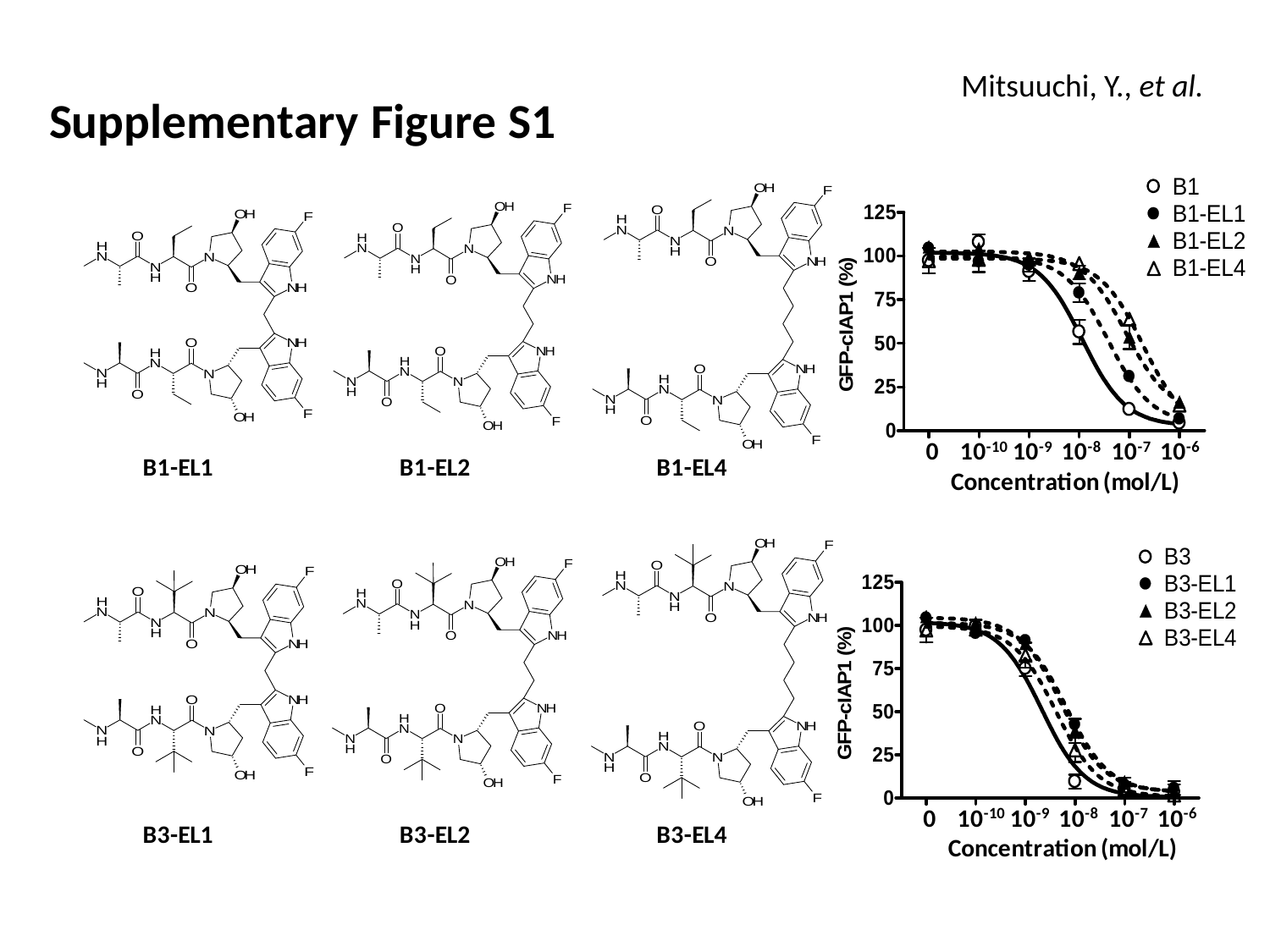

Mitsuuchi, Y., et al.

Supplement: Supplementary Figure 1 [file cddiscovery201646-s1.ppt]

## Slide 1
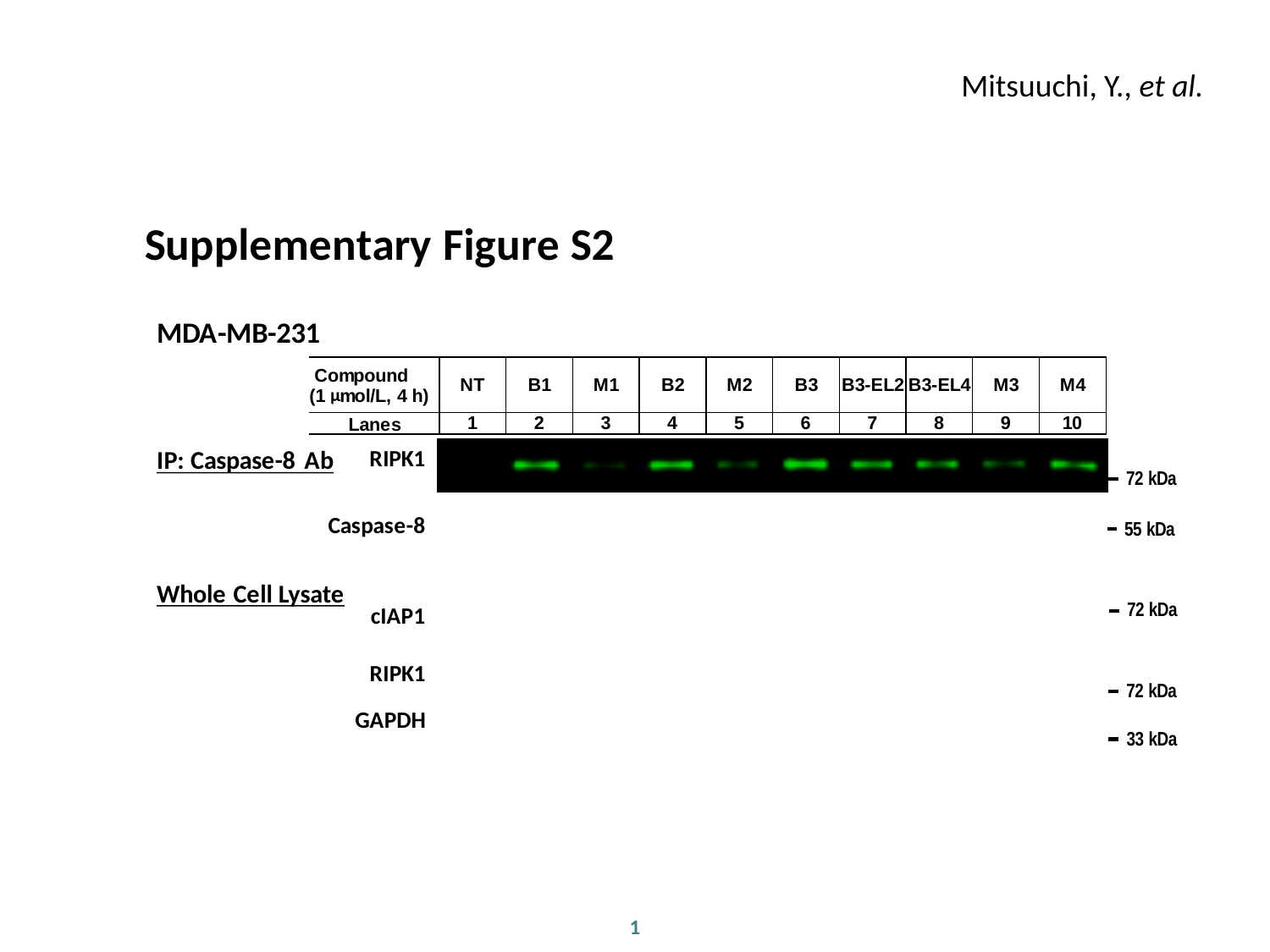

Mitsuuchi, Y., et al.
<number>

Supplement: Supplementary Figure 2 [file cddiscovery201646-s2.ppt]

## Slide 1
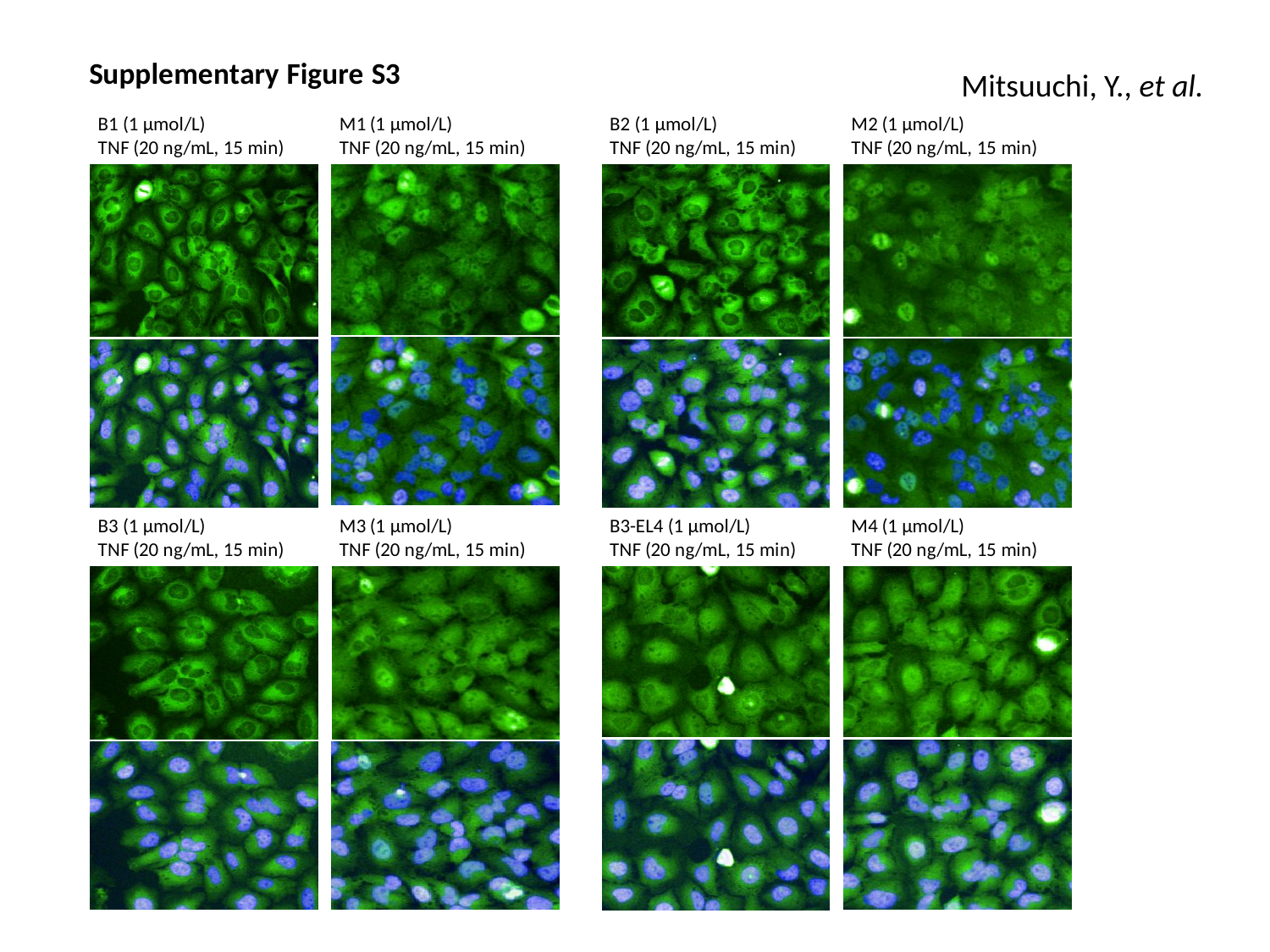

Mitsuuchi, Y., et al.

Supplement: Supplementary Figure 3 [file cddiscovery201646-s3.ppt]

## Slide 1
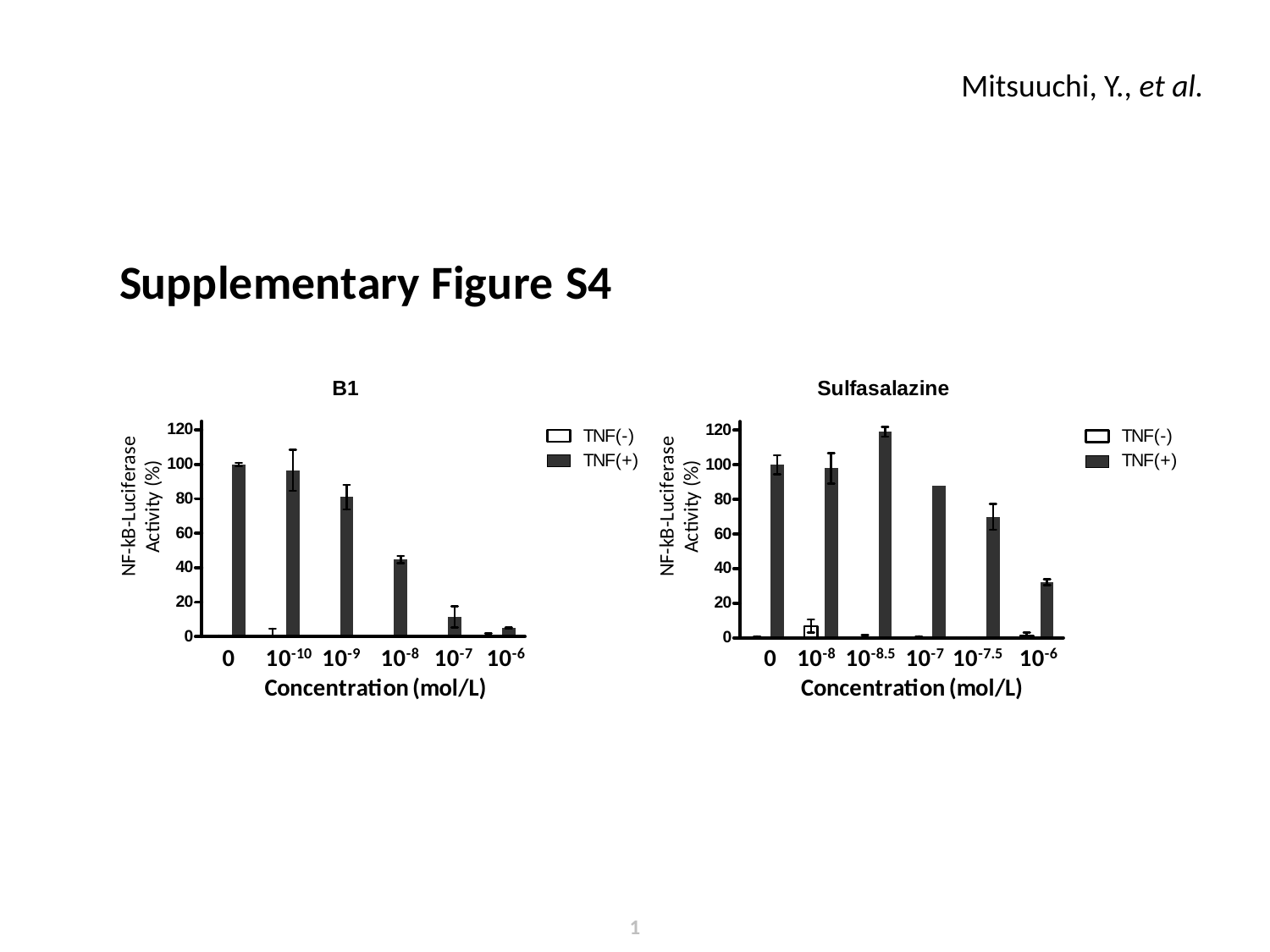

Mitsuuchi, Y., et al.
<number>

Supplement: Supplementary Figure 4 [file cddiscovery201646-s4.ppt]
