# Supplementary material for: Bivalent IAP antagonists, but not monovalent IAP antagonists, inhibit TNF-mediated NF-κB signaling by degrading TRAF2-associated cIAP1 in cancer cells
Source: Cell Death Discov. 2017 Jan 16;3:16046–. doi: 10.1038/cddiscovery.2016.46 (PMC5238498; doi:10.1038/cddiscovery.2016.46)
Supplement: Supplementary Figures Legend [file cddiscovery201646-s5.doc]

**SUPPLEMENTAL FIGURE LEGENDS**

**Supplementary Figure S1.**

Chemical structures of the linker chain-extended bivalent IAP antagonists. B1 and B3 were modified by inserting a methylene (EL1), ethylene (EL2), or *n*-butylene (EL4) chain at P4 position and evaluated by GFP-cIAP1 degradation assay (results show mean +/- SE, n=5 experiments).

**Supplementary Figure S2.**

Both monovalent and bivalent IAP antagonists promoted RIPK1:caspase-8 death protein complex formation. IAP antagonist-sensitive breast cancer cell line, MDA-MB-231, was evaluated for RIPK1:caspase-8 complex formation. Following IAP antagonist treatment, cell lysates were subjected to IP with anti-caspase-8 antibody, and the RIPK1:caspase-8 complex was evaluated by the Western blot analysis with anti-RIPK1 antibody. Representative result from 2 independent experiments.

**Supplementary Figure S3.**

Bivalent IAP antagonists inhibited the nuclear translocation of p65/NF-κB induced by TNF stimulation in HeLa cells. p65/NF-κB was detected by using Alexa Fluor®-conjugated anti-p65/NF-κB antibody. The nuclear DNA was counter-stained with Hoechst33342: the blue nuclear stain is shown in each of the bottom panels. Fluorescence images were captured by using Operetta® High Content Imaging System.

**Supplementary Figure S4.**

B1 and Sulfasalazine showed comparable activity in inhibition of the transcriptional activity by the p65/NF-κB reporter gene assay (n=2). HeLa cells stably harboring p65/NF-κB reporter gene were pretreated with B1 or Sulfasalazine 2 h prior to TNF (20 ng/mL) stimulation. Luciferase activity was measured as described in Materials and Methods. Results show mean +/- SD.
